# Palladium doped PDA-coated hercynite as a highly efficient catalyst for mild hydrogenation of nitroareness

**DOI:** 10.1038/s41598-024-62226-5

**Published:** 2024-05-25

**Authors:** Somaye Beheshti, Alireza Motavalizadehkakhky, Rahele Zhiani, Seyed Mohammad Mahdi Nouri, Ehsan Zahedi

**Affiliations:** 1 Department of Chemistry, Neyshabur Branch, Islamic Azad University, Neyshabur, Iran; 2 Avdanced Research Center for Chemistry, Biochemistry and Nanomaterial, Neyshabur Branch, Islamic Azad University, Neyshabur, Iran; 3 New Materials Technology and Processing Reserearch Center, Neyshabur Branch, Islamic Azad University, Neyshabur, Iran; 4 Chemical Engineering Department, Hakim Sabzevari University, Sabzevar, Iran; 5 Department of Chemistry, Shahrood Branch, Islamic Azad University, Shahrood, Iran

**Keywords:** Hercynite, Grafting, Magnetic nanoparticles, Nanocatalyst, Reduction reaction, Chemistry, Catalysis, Inorganic chemistry, Organic chemistry, Surface chemistry, Chemical synthesis

## Abstract

Hercynite magnetic nanoparticles were produced through the co-precipitation of ferrous and aluminum cations. The surface of hercynite was respectively coated with silica, 2,4,6-trichloro-1,3,5-triazine, and 1*H*-pyrazole-3,5-dicarboxylic acid to provide a suitable substrate for Pd(II) loading, furnishing Pd@Her-TCT-PDA. Subsequently, the introduced Pd(II) was reduced to Pd(0) using NaBH_4_. FT-IR, EDS, XRD, TGA, TEM and SEM images were the characteristic methods to prove the success of catalyst synthesis. The SEM image illustrated the particles with a nanosize of 25–50 nm and TEM image confirmed the presence of Pd nanoparticles with sizes lower than 2 nm. EDS elemental analysis of the catalyst proved the existence of Pd, Fe, and Al atoms along with the C, O, N, and Si atoms belong to the heterocyclic moieties. VSM analysis clarified a considerable drop in the magnetic properties of the hercynite core of the final catalyst due to its modified surface. TGA curve demonstrated that Pd@Her-TCT-PDA contains 20% organic content, attributed to the anchored heterocyclic ligands. Finally, Pd@Her-TCT-PDA was employed along with NaBH_4_ as a catalytic system to reduce completely the nitro group of aromatic compounds to their corresponding amines. The recyclability tests showed low drop in the catalytic activity of Pd@Her-TCT-PDA after third run with negligible leaching of Pd NPs.

## Introduction

**Figure 1:**
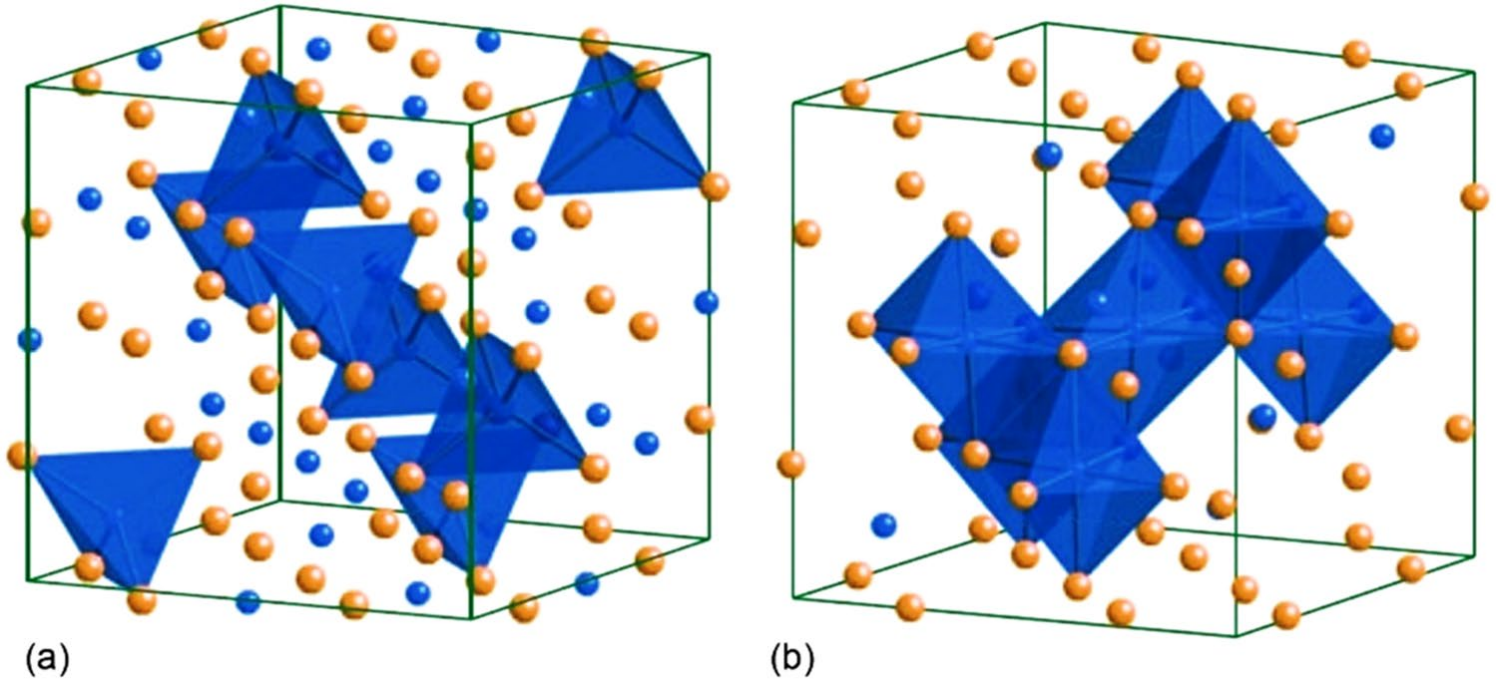
The structure of (**a**) tetrahedral spinel, (**b**) octahedral spinel around cations31.

The spinel compounds with a general formula of AB_2_X_4_ are a category of minerals with special characteristics resulting from their cubic, isomeric crystal system, including high corrosion resistance and exceptional optical and mechanical properties. Because of their remarkable properties, spinels have extensive applications in various fields of magnetism, electronics, storage and transformation of energy, and catalysts^1–6^. Hercynite (FeAl_2_O_3_) has a tetrahedral structure, in which the distribution of ferrous and aluminum cations in its network is such that one eighth of sites are occupied by ferrous cations and aluminum cations occupy half of these sites (Fig. 1)^7–11^. Among the known spinels, hercynite is the most expensive one because of its magnetic properties resulting from ferrous ions, high chemical stability, and great mechanical behavior^12,13^.

Recently, hercynite has become a good candidate to make novel magnetic catalysts due to its large surface area, high capacity for adsorption of chemicals, magnetic character, and considerable physicochemical stability^14,15^. Moreover, it is synthesizable in the laboratory through various methods, including reaction sintering^8^, chemical vapor deposition^12^, microwave magnetic field (H-field) irradiation^16^, combustion^17^, molten salt^18^, co-precipitation, and sol–gel methods^19^. Due to its magnetic properties, hercynite is extensively considered as the magnetic core of a catalyst^20^, and the hydroxyl groups on its surface are suitable to be modified with various catalytic species^21^. Consequently, a wide variety of homogeneous catalysts can be incorporated on the surface of hercynite to create a heterogeneous, magnetic catalyst, which can be separated using a magnet from the reaction mixture^22–24^. So far, hercynite-based catalysts have been employed in the solar thermochemical hydrogen production^22,25^, production of bioactive heterocycles^26–28^, and detoxification of aflatoxin B1^29^.

Aniline is used in the production of many chemicals and pharmaceuticals, herbicides, dyes, and antioxidants, thus the reduction of nitrobenzene to aniline a valuable industrial reaction^31^. Hydrogenation of nitrobenzene is one of the best reductive ways of industrial aniline synthesis^32–34^, which can be performed through transfer hydrogenation using reducing agents, direct hydrogenation under high pressure, and photocatalytic hydrogenation^32,35,36^. Since reduction of nitro compounds is conducted using expensive metal catalysts, including Pd/C, Pt(IV) oxide, or Raney nickel, isolating and reusing these catalysts is crucial for large-scale processes^37–41^. On the other hand, selectivity of hydrogenation and running the reaction under green conditions are also considered as the main factors of catalyst selection^42,43^. Carrying forward our earlier research on devising innovative nanocatalysts^44–48^ and developing organic syntheses^49–51^, herein, we wish to investigate the reductive conversion of nitro groups of aromatic organic compounds to their corresponding amines using a heterogenized palladium catalyst. In this regard, we prepared a nanosized hercynite and modified its surface with silica and organic ligands to be suitable for the loading of palladium ions. Thus, the loaded palladium can reduce the nitro compounds, then it is readily isolated using a magnet and reused. Using this approach, the recovery and recyclability of the precious Pd catalyst are improved.

## Experimental

### Materials and instruments

All chemical used in this project were prepared in analytical grade with high purity from Sigma-Aldrich Company which are included metal salts of FeCl_2_.4H_2_O and Al(NO_3_)_3_.9H_2_O, silicon-based materials of tetraethyl orthosilicate (TEOS) and (3-aminopropyl)triethoxysilane (APTES), heterocycles of 2,4,6-trichloro-1,3,5-triazine (TCT), 1*H*-pyrazole-3,5-dicarboxylic acid (PDA), morpholine, and pipyrazine, Pd(OAc)_2_, phenylacetylene, NaBH_4_, aminobenzenes, various aromatic aldehydes, toluene, EtOH, THF, and ammonia solution (NH_4_OH, 25%).

The crystalline and phase structure of the Her-TCT-PDA nanocomposite was characterized by various analytical techniques, including X-ray diffraction (XRD), Siemens, Germany, model of D5000 equipped with Cu-Kα radiation over a range of 2θ 10–90°. Fourier transforms infrared (FT-IR) spectra were recorded between 4000 and 400 cm^−1^ on a BRUKER, Germany, EQUINOX 55, and using KBr pellet. Field emission scanning electron microscopy (FESEM) images along with an energy dispersive spectroscopy (EDS) analysis were obtained using a MIRA3 Tescan instrument, Czech Republic, with an acceleration voltage of 20 kV. To measure the loading and leaching amounts of palladium ions on/from the catalyst surface, an inductively coupled plasma of ICP-AES using Vista-pro device, Agilent (HP), California, USA, was employed. Transmission electron microscopy (TEM) of Philips CM30300Kv instrument, Germany, was used to obtain morphology images, while the samples were deposited on a carbon coated copper grid.

### General procedure: synthesis of Pd/Her-TCT-PDA

#### Synthesis of hercynite (FeAl_2_O_4_)

A chemical co-precipitation process was the way for the preparation of FeAl_2_O_4_.^30^ Initially, FeCl_2_.4H_2_O (3.01 mmol, 0.0965 g) and Al(NO_3_)_3_·9H_2_O (1.50 mmol, 0.562 g) with a molar ratio of 2:1 were dissolved in warm water (100 mL, 80 °C) under N_2_ atmosphere. Then, NaOH solution (15 mL, 0.2 M) was poured into the mixture gradually for 5 min so that the pH of the solution reached 12. The precipitate of hercynite (FeAl_2_O_4_) MNPs was collected using an external magnet and washed well with deionized water. The obtained hercynite MNPs was dried at 75 °C overnight.

#### Synthesis of Her@SiO_2_

The Stöber method was employed to modify the surface of hercynite. Correspondingly, hercynite (8.6 mmol, 1.5 g) was dispersed in distilled water (16 mL) in an ultrasonic bath, then mixed with NH_3_ solution (2 mL, 25 wt%) and EtOH (80 mL). Subsequently, TEOS (2.6 mL) was slowly added to the hercynite solution under intense stirring at ambient temperature. When TEOS was completely added, the mixture was continuously stirred at room temperature for 24 h. The product was lastly separated by an external magnet, washed well with distilled water, and dried at 50 °C.

#### Synthesis of Her-NH_2_

The dried Her@SiO_2_ MNPs was dispersed in dry toluene (50 mL) via sonication for 30 min. Then, APTES (5 mL) was gradually added under an inert (N_2_) atmosphere and refluxed overnight at 110 °C. Upon completion of the reaction, the functionalized hercynite was separated, washed with dry toluene several time for removal of the remaining APTES, and dried at 100 °C overnight.

#### Synthesis of Her-TCT

To incorporate the TCT molecules on the surface of Her-NH_2_, a mixture of TCT (6.7 mmol, 1.24 g) and Her-NH_2_ (1.5 g) in THF (50 mL) was stirred in an iced-bath. Subsequently, trimethylamine (1 mL) was added to it dropwise. Upon completing the addition of the latter, the mixture was stirred at room temperature for 24 h. Finally, the precipitate was filtered off and washed with THF repeatedly. The obtained Her-TCT was dried for 12 h at 70 °C.

#### Synthesis of Her-TCT-PDA

Her-TCT (1.2 g) was dispersed in dry toluene (40 mL) and PDA (12.8 mmol, 2 g) was poured into the mixture and refluxed under N_2_ atmosphere for 24 h. Afterward, the solid was filtered off and washed with hot EtOH three times. The obtained Her-TCT-PDA dried for 12 h at 70 °C.

#### Synthesis of Pd/Her-TCT-PDA

**Scheme 1:**
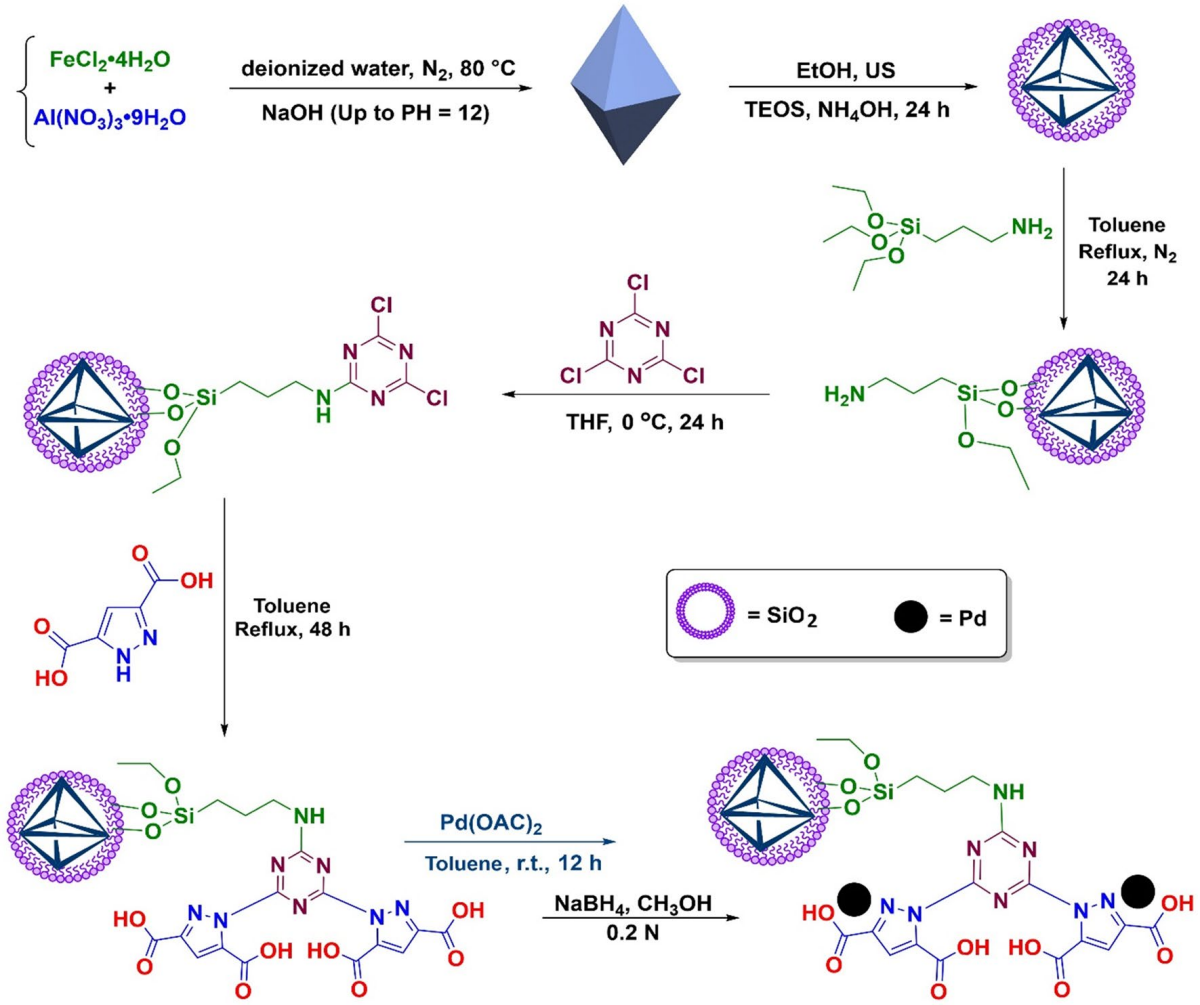
The schematic route of Pd@Her-TCT-PDA preparation.

To incorporate Pd ions on the surface of Her-TCT-PDA, a mixture of Pd(OAc)_2_ (0.1 mmol, 0.02 g) and Her-TCT-PDA (1.2 g) suspended in dry toluene (15 mL) was stirred at ambient temperature for 10 h. The reduction of Pd(II) to Pd(0) was then performed through the gradual addition of a solution of NaBH_4_ in methanol (10 mL, 0.2 N) to the Pd(II)@Her-TCT-PDA mixture. Then, the resulting mixture was vigorously stirred for 5 h to complete the reduction process. Next, the solid of Pd@Her-TCT-PDA was filtered, washed well with cold MeOH, and dried in an oven at 80 °C overnight. The schematic process of synthesis of the catalyst is illustrated in Scheme 1.

### Hydrogenation reaction

**Scheme 2:**
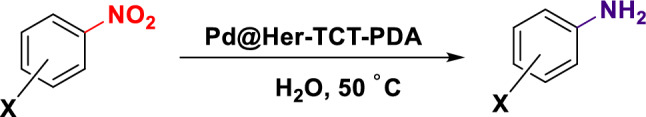
Hydrogenation of nitro arenes.

Nitro aromatic compound (1 mmol), 5 mL water, and 0.04 g of Pd@Her-TCT-PDA catalyst was vigorously stirred in a round-bottomed flask at room temperature. Then NaBH_4_ (2 mmol, 0.076 g) was added, and the reaction was warmed up to 50 °C (Scheme 2). Completion of the reduction was traced by TLC, then, the catalyst was separated by an external magnet. The mixture was cooled down, and the precipitate was filtered off and recrystallized from EtOH to give the pure product.

## Result and discussion

### Catalyst preparation

The schematic process of synthesis of the Pd@Her-TCT-PDA catalyst is illustrated in Scheme 1. Initially, hercynite (*Her*, FeAl_2_O_4_) nanoparticles was prepared through the reaction between ferrous chloride and aluminum nitrate. Then, the surface of Her MNPs was coated with silica to have an inert, modifiable surface to react with APTES. Subsequently, the amine-functionalized Her (Her-NH_2_) was treated with TCT heterocycle, followed by PDA, to have a suitable substrate for the incorporation of palladium ions. Finally, the loaded Pd(II) ions were reduced to Pd(0) through the reaction of Pd@Her-TCT-PDA with NaBH_4_ solution. To confirm the effectiveness of this procedure, the target solid Pd(0)@Her-TCT-PDA was characterized using various analytical techniques.

### Catalyst characterization

**Figure 2:**
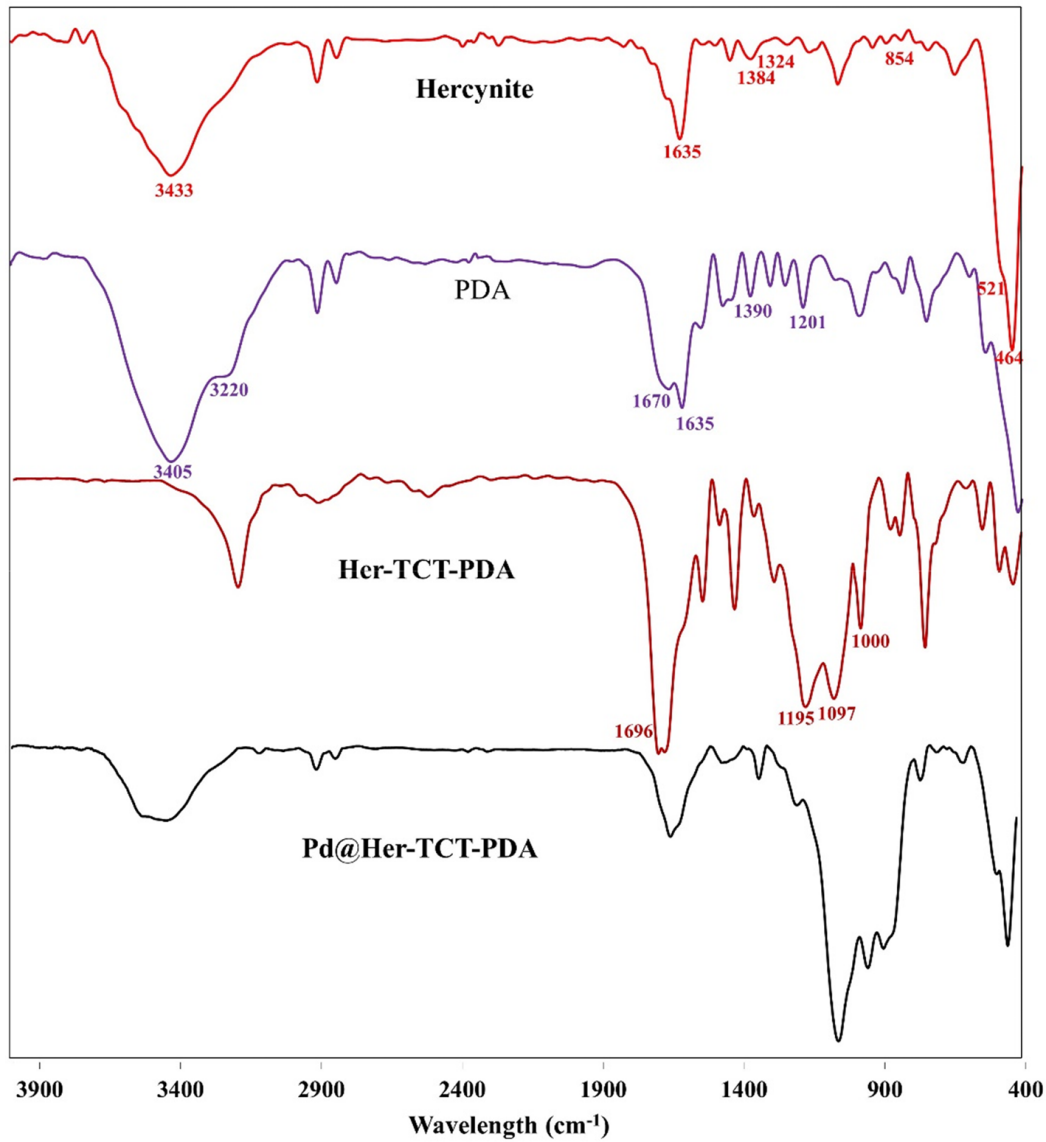
The FTIR spectra of (**a**) hercynite, (**b**) PDA, (**c**) Her-TCT-PDA, and (**d**) Pd@Her-TCT-PDA.

The FT-IR spectra of hercynite, PDA, Her-TCT-PDA, and Pd@Her-TCT-PDA nanocomposite are compared in Fig. 2a–d, respectively. The spectrum of hercynite MNPS (Fig. 2a) shows two main bands at 464 and 521 cm^−1^, which are attributed to the Fe–O vibration^52^. A weak vibration related to the Al-O bonds is observable around 854 cm^−1^. Furthermore, the band at 3433 cm^−1^ is due to O–H vibrations on the surface of hercynite^53^. The appeared bands at 1324, 1384, and 1635 cm^−1^ are as a result of the remained nitrate impurities in the hercynite structure^54^. In the case of 3,5-pyrazoledicarboxylic acid (PDA, Fig. 2b), the O–H stretching vibration of its carboxylic acid groups are observed around 3405 cm^−1^. The NH stretching vibration of PDA gave a absorbance band at 3220 cm^−1^. The characteristic stretching vibrations of C=O of carboxylate groups are apparent as two strong bands at 1670 and 1635 cm^−1^, while various N–N, C–N, and N–H vibrations are seen between 1201 and 1390 cm^−1^. The FT-IR spectrum of Her-TCT-PDA is also depicted in Fig. 2c. The main absorbance bands of PDA and Her spectra appear in this spectrum, although the wide band for hydrogen bonds is vanished due to the deep removal of moisture. In fact, the main distinguished band at 1696 cm^−1^ is related to –C=N of TCT. Furthermore, the sharp bands at 1000, 1097, and 1195 cm^-1^ correspond to the Si–O–Si vibration of the silica layer. The FT-IR spectrum of Pd@Her-TCT-PDA is shown in Fig. 2d. It could be seen that after the deposition with Pd ions on Her-TCT-PDA, there was a little change in the FT-IR spectrum of the prepared nanocatalyst, due to the complexation between ligands and Pd ions. However, the latter indicates that the Her-TCT-PDA remained stable during the incorporation of the palladium ions.

**Figure 3:**
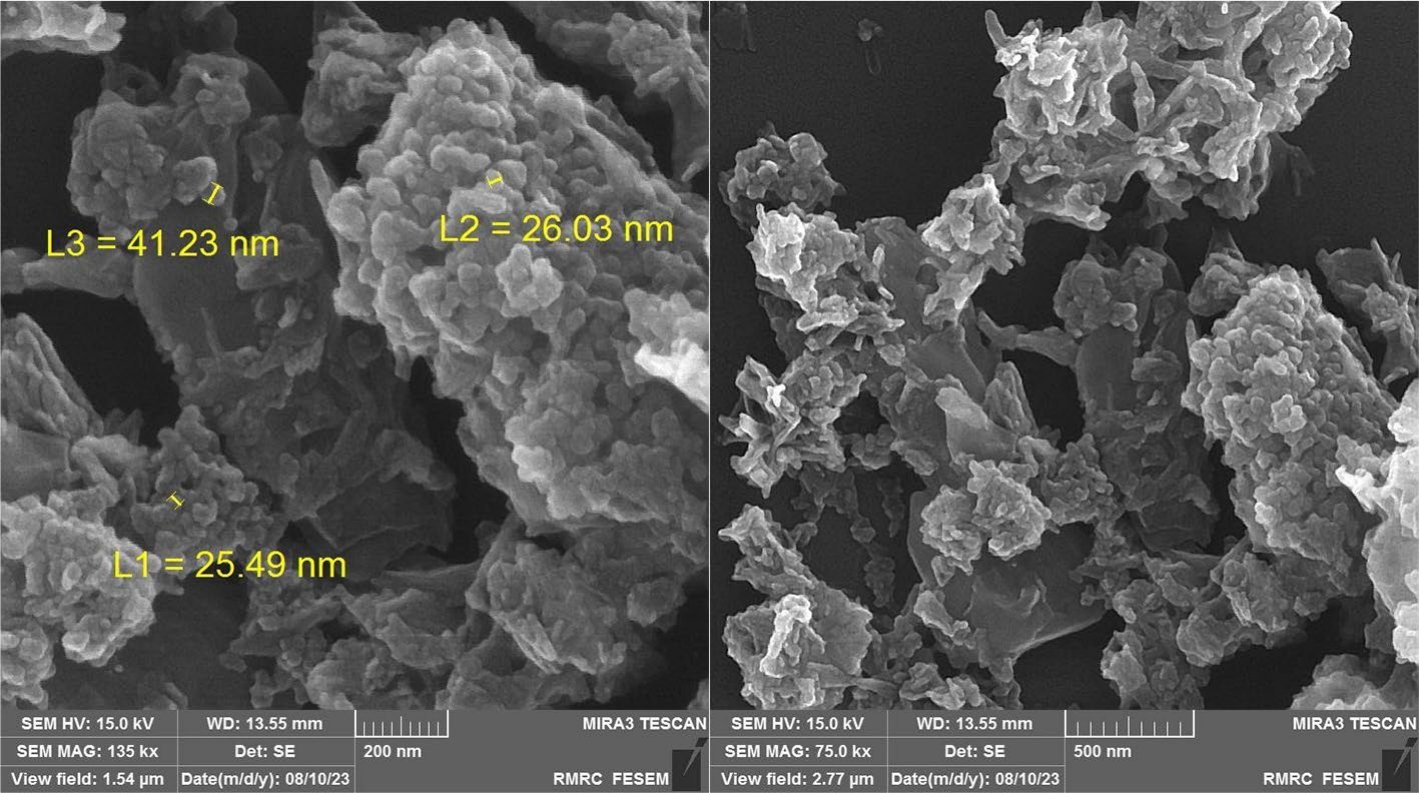
SEM images of Pd@Her-TCT-PDA.

**Figure 4:**
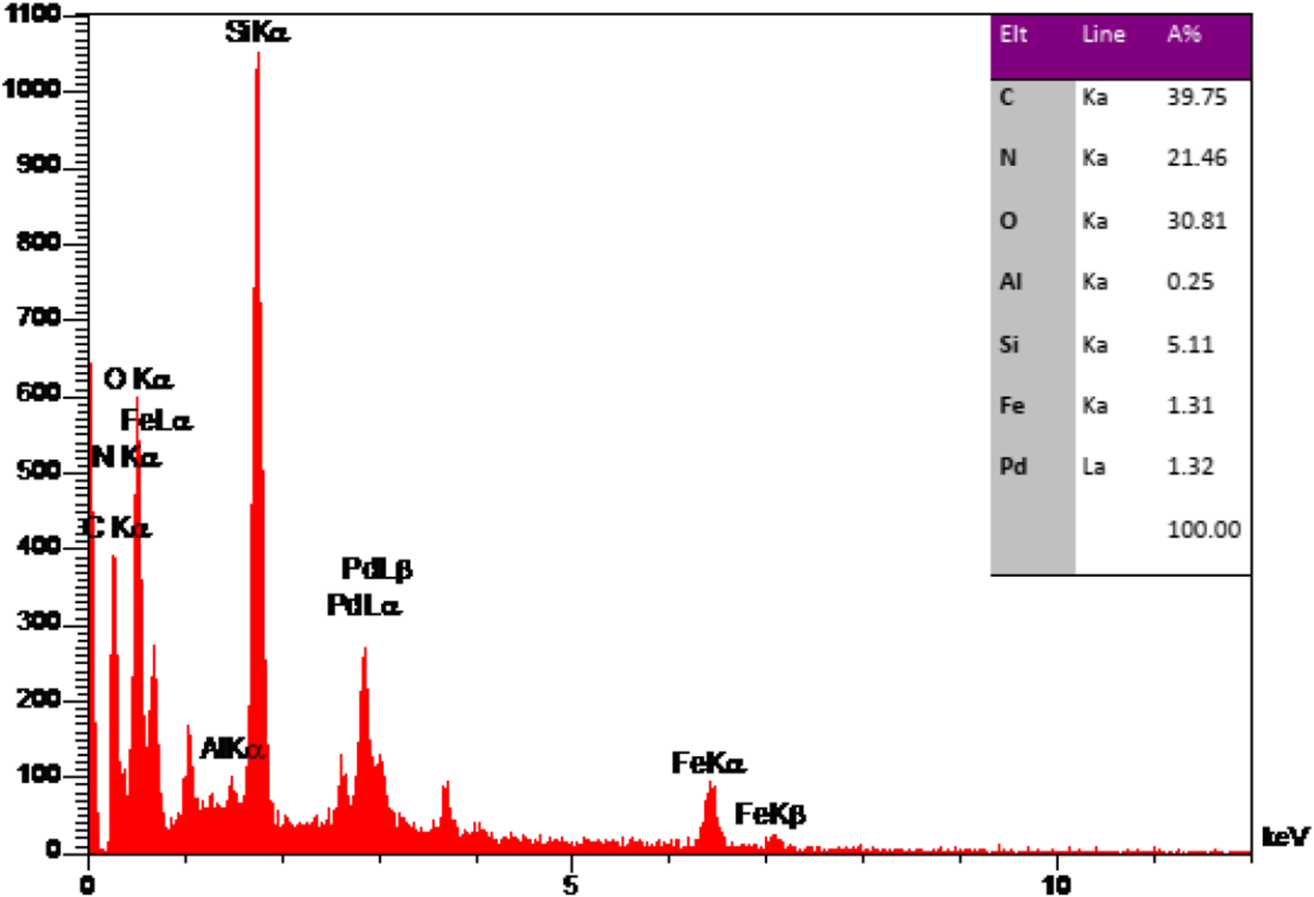
EDX analysis of images of Pd@Her-TCT-PDA.

**Figure 5:**
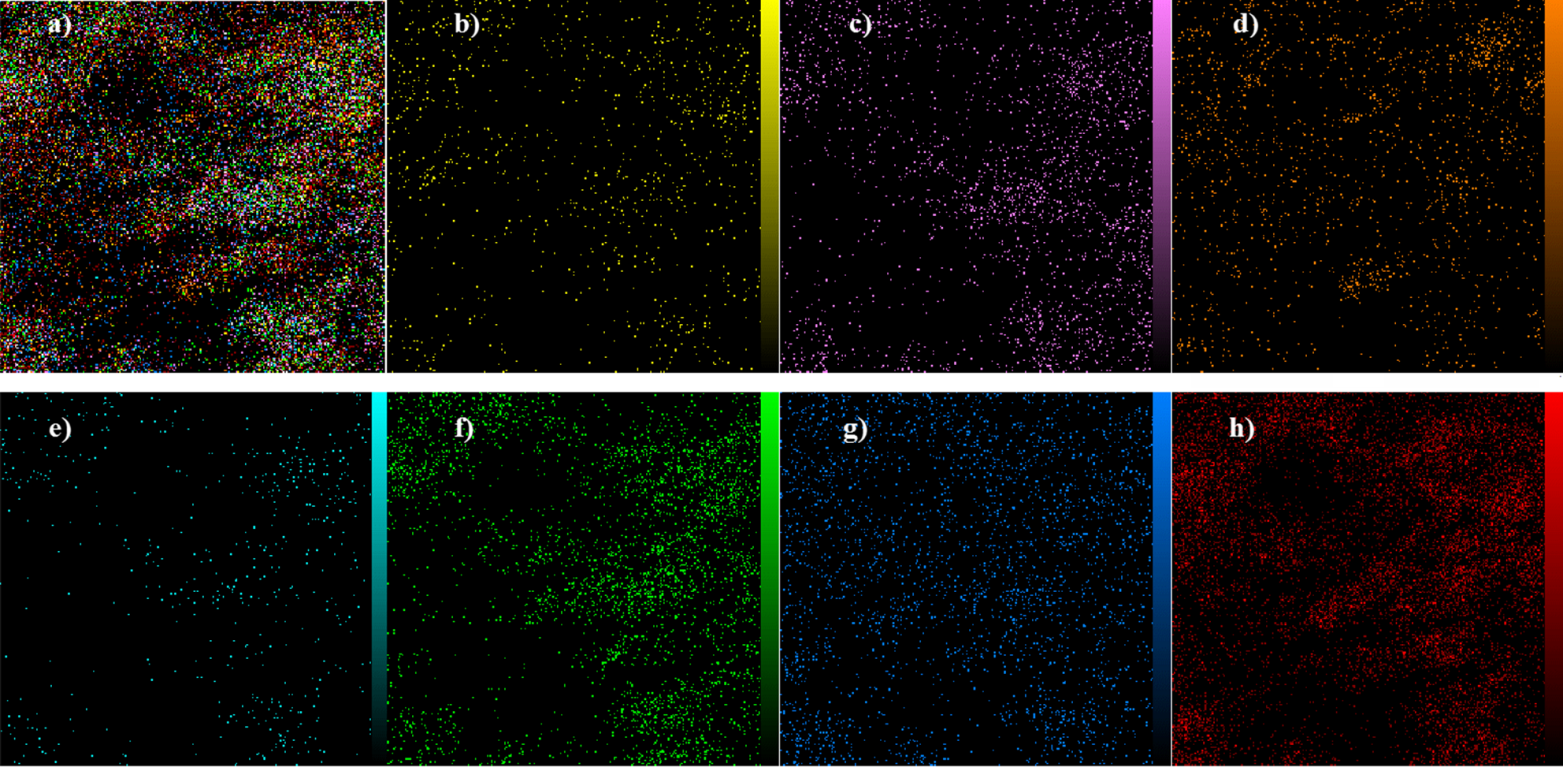
EDX-mapping analysis of Pd@Her-TCT-PDA nanocatalyst. (**a**) combine, (**b**) Aluminum (Ka), (**c**) Carbon (Ka), (**d**) Iron (Ka), (**e**) Nirogen (Ka), (**f**) Oxygen (Ka), (**g**) Palladium (La), (**h**) Silicone (Ka).

The morphology and elemental characteristics of the Pd@Her-TCT-PDA were studied through SEM and EDS techniques, respectively (Figs. 3–5). The SEM images of Pd@Her-TCT-PDA (Fig. 3) demonstrate that most of the synthesized nanoparticles have a uniform spherical shape with an average size of 25–42 nm. The EDS analysis of the Pd@Her-TCT-PDA catalyst (Fig. 4) displays the presence of Si, Al, Fe, O atoms which exist in the structure of silica coated hercynite. The appearance of C, N, and O atoms approves the incorporation of TCT and PDA. The X-ray mapping of Pd@Her-TCT-PDA MNPs (Fig. 5) indicates good dispersion of Fe, Al, C, N, O, and Pd atoms in the scaffold of the catalyst, confirming the uniform coating of hercynite with silica and the excellent incorporation and loading of heterocycles and palladium on its surface.

**Figure 6:**
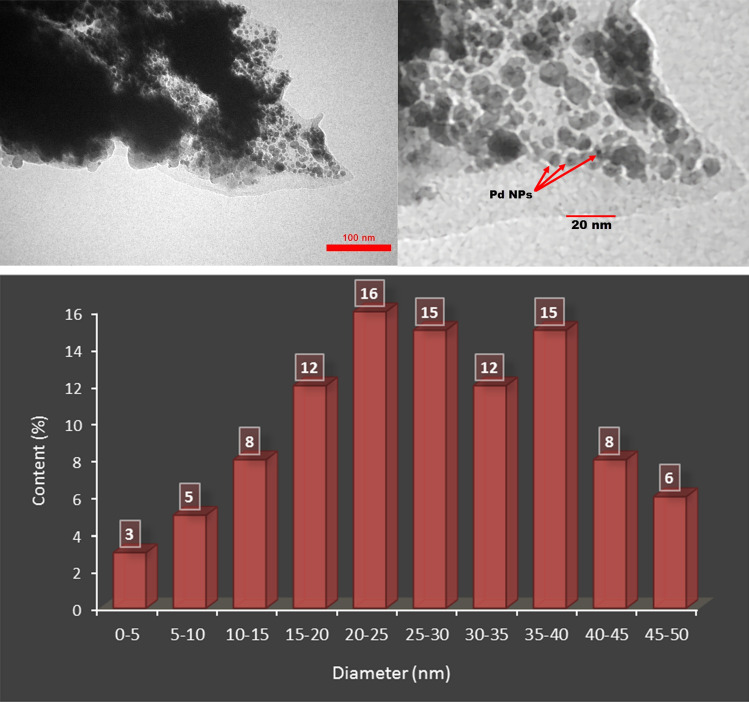
Up) TEM images of Pd@Her-TCT-PDA. Down) Histogram of particles size.

As shown in Fig. 6, the TEM images show a spherical shape morphology for MNPs Pd@Her-TCT-PDA as well as some aggregation is observed. According to this image, the size of Pd NPs is about 1–2 nm. Moreover, Fig. 6 shows the histogram of particles size, confirming the presence of particles with diameters lower than 5 nm that are attributed to the palladium particles.

**Figure 7:**
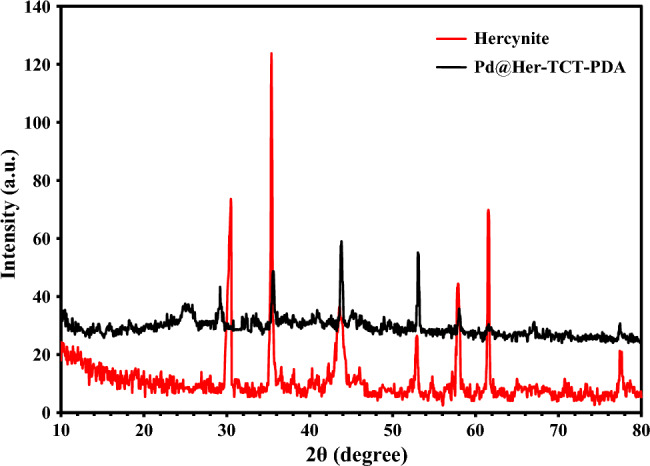
XRD pattern of hercynite and Pd@Her-TCT-PDA.

The X-ray diffraction (XRD) patterns of hercynite (Fig. 7, orange pattern) depict the sharp diffraction peaks that correspond to the FeAl_2_O_4_ MNPs (ICSD name: Iron Aluminum Oxide), with high crystallinity, confirming its cubic structure because of the sharp peaks appeared at 2θ values of 30.48, 35.38, 43.63, 57.93, 61.58, and 77.58°^11^. Moreover, Pd@Her-TCT-PDA (Fig. 7, blue pattern) exhibits six main distinctive peaks at 2θ values of 25.30, 35.63, 43.83, 53.09, 57.98, and 77.38°, which are attributed to the standard XRD pattern of hercynite. On the other hand, it can be concluded that the crystalline phase of hercynite MNPs didn’t change during coating with silica and chemical modification. Moreover, according to the JCPDS card number of 46–1043, the appearance of peaks at 2θ equal to 39.69, 45.68 and 65.94° can confirm the presence of Pd(0)^7,55^. Fig. 7 shows sharp peaks for FeAl_2_O_4_ MNPs, while the mentioned peaks for Pd(0) is not observable within the low intensities peaks because the concentration of Pd particles on the catalyst surface is lower than the FeAl_2_O_4_ MNPs.

**Figure 8:**
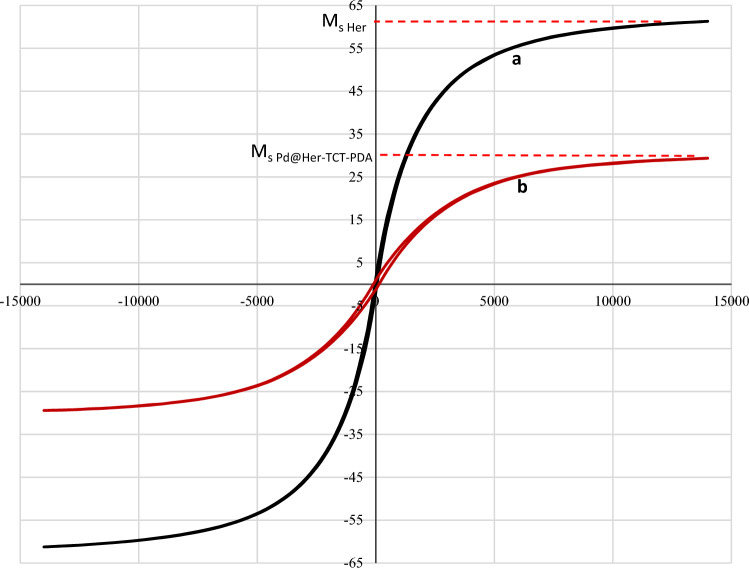
VSM analysis of (**a**) hercynite, (**b**) Pd@Her-TCT-PDA.

To study the magnetic properties of coated hercynite with inorganic/organic chemicals, a vibrating-sample magnetometer (VSM) of Pd@Her-TCT-PDA was determined at ambient temperature (Fig. 8). The obtained M_s_ value for Pd@Her-TCT-PDA was about 29.91 emu/g, showing a considerable drop in comparison with that of hercynite (60.85 emu/g). These results demonstrated that the incorporation of nonmagnetic components can distinctly decrease the magnetic property of hercynite. Although the hysteresis loops of Pd@Her-TCT-PDA exhibited a superparamagnetic behavior with their dispersion stability in solution without aggregation^7^.

**Figure 9:**
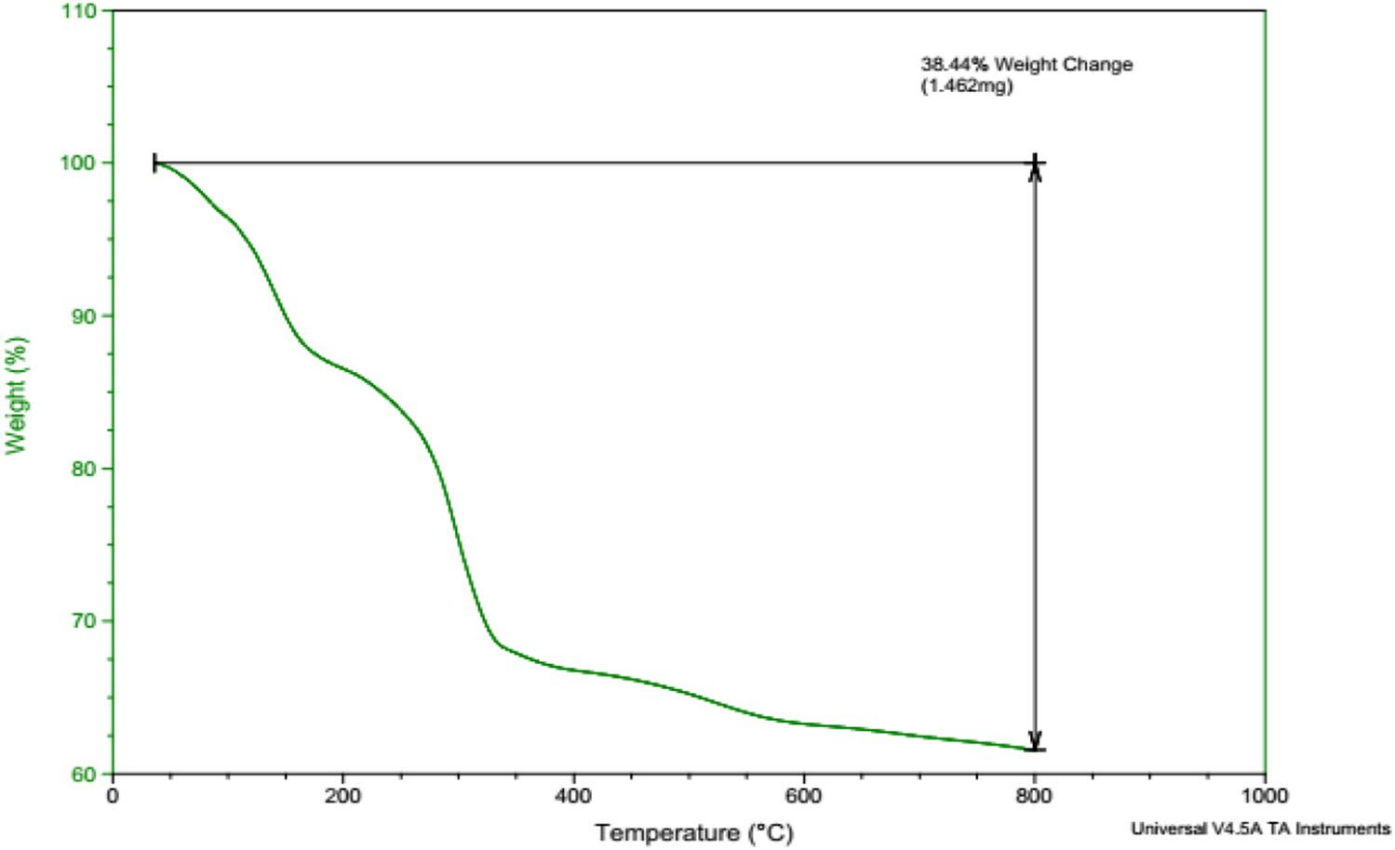
TGA analysis of Pd@Her-TCT-PDA.

Thermogravimetric analysis (TGA) of Pd@Her-TCT-PDA MNPs was applied to survey the thermal stability of the immobilized TCT and PDA on hercynite and its weight loss (Fig. 9). The initial 10% mass loss of the TGA curve before 200 °C is due to the removal of adsorbed organic solvents and moisture on the surface of the MNPs. So, the subsequent weight loss (20%) after 200 °C to and 350 °C indicates the decomposition of organic moieties such as TCT, PDA, and propyl amine groups. The third mass loss (6%) is due to the condensation of surface hydroxyl groups and the oxidative decomposition of volatile products from the immobilized amorphous phase of the silica segment.

**Scheme 3.**
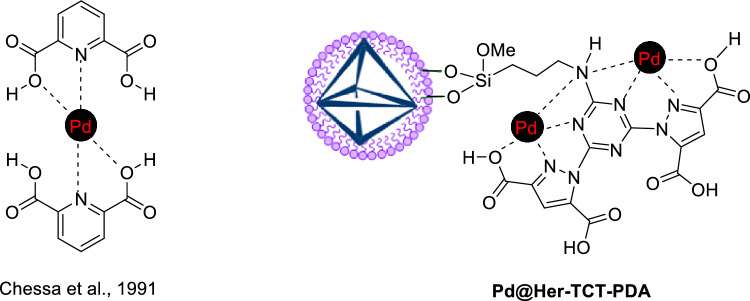
The probable complex of Pd@Her-TCT-PDA.

According to the published articles, palladium forms a complex with nitrogen and oxygen atoms of dipicolinic acid (pyridine dicarboxylic acid), as depicted in Scheme 3. The complexation has been confirmed by crystallographic analysis^56–58^. Therefore, the neighborhood of carboxylates’ oxygen atoms with nitrogen atoms of pyrazole moieties in Pd@Her-TCT-PDA raises the possibility that palladium ions are attached to the ligands through their oxygen and nitrogen atoms, as depicted in Scheme 2.

### Catalytic study

The reduction of nitroaromatic compounds was the way to examine the catalytic activity of Pd@Her-TCT-PDA. As a model reaction (Table 1), the reduction of nitrobenzene was studied at different reaction times, catalyst amounts, and several solvents, including water, dimethylformamide (DMF), acetonitrile (MeCN), ethanol (EtOH), and dichloromethane (CH_2_Cl_2_). Initially, the reduction of nitro group was performed in different solvents to find the best one (entries 1–5). The results indicated that the use of protic, polar solvent, i.e. EtOH and water, can remarkably increase the reaction yield. Accordingly, running the reaction in water at 50 °C converted the nitro group to amine completely. Accomplishment of the reaction at room temperature led to a considerable drop in conversion. Moreover, the effect of catalyst amounts was tested (entries 9 and 10). Consequently, to find the general applicability of Pd@Her-TCT-PDA in the reduction of nitro groups, 0.04 g of this catalyst was used in the reduction of various nitro compounds in warm water (50 °C).

**Table 1:** Optimization conditions for reduction of nitrobenzene.


Entry	Catalyst amount (g)	Solvent	Temp. (°C)	Time (min)	Yield (%)	TON^1^	TOF^2^
1	0.04	MeCN	50	40	10	30	0.76
2	0.04	CH_2_Cl_2_	reflux	45	trace	–	–
3	0.04	DMF	50	40	10	30	0.76
4	0.04	EtOH	50	30	70	212	7.1
5	0.04	H_2_O	50	35	100	303	8.6
6	0.04	H_2_O	70	38	99	300	7.9
7	0.04	H_2_O	reflux	35	99	300	8.6
8	0.04	H_2_O	r.t	45	45	136	3.0
9	0.03	H_2_O	50	30	95	380	12.6
10	0.05	H_2_O	50	30	100	238	7.9

Conditions: Nitrobenzene (1 mmol, 0.12 g), NaBH_4_ (2 mmol, 0.076 g), solvent (5 mL).

^1^TON: turnover number was calculated as the number of moles of amine per moles of Pd.

^2^TOF: turnover frequency was calculated as TON per minutes.

Table 2 represents the obtained results that confirm the exceptional catalytic performance of Pd@Her-TCT-PDA MNPs in the reduction of nitro group to amine, while complete conversions were achieved at short reaction times.

**Table 2:** Reduction of nitro compounds in the presence of Pd@Her-TCT-PDA and NaBH_4_.

Entry	Product	Time (min)	Yield (%)	Melting point (Abs./Lit.) [1,2]	TON^1^	TOF^2^ (min^−1^)
1		30	100	143–144/145–147	300	10
2		34	97	100–103/102–104	294	8.6
3		35	99	Liquid	300	8.57
4		40	97	59–61/60–64	294	7.4
5		40	95	46/45–46	288	7.2
6		38	98	56–58/58	297	7.8
7		45	94	48–51/50	285	6.3
8		38	98	72–74/ 73	297	7.8
9		45	90	101–102/100–105	273	6.1
10		50	92	187–189/186–190	279	5.6
11		35	92	76–77/77–79	279	8.0
12		35	92	28–31/28–30	279	8.0
13		120	94	184–187/187–189	284	2.37
14		40	96	Liquid	291	7.3

Conditions: Nitro-compound (1 mmol, 0.12 g), NaBH_4_ (2 mmol, 0.076 g), catalyst (0.04 g), and H_2_O (5 mL) mixing at room temperature.

^1^TON: turnover number was calculated as the number of moles of amine per moles of Pd.

^2^TOF: turnover frequency was calculated as TON per minutes.

### Reaction mechanism

**Figure 10:**
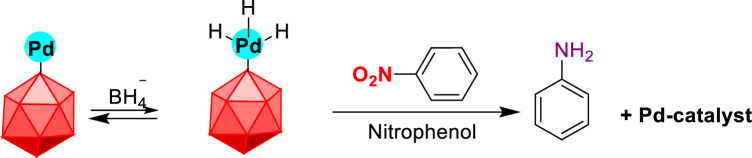
The plausible mechanism for the reduction of nitrobenzene on the Pd@Her-TCT-PDA nanocatalyst.

The mechanism of catalytic reduction of nitrobenzene to aniline in the presence of Pd@Her-TCT-PDA MNPs can be described by the Langmuir–Hinshelwood model^59,60^. Correspondingly, the hydride ions (H^–^) of the adsorbed BH_4_^¯^ on the nanocatalyst surface are transferred to the Pd NPs surface (Fig. 10). Afterwards, by approaching nitrobenzene to the Pd on the catalyst surface, interfacial electron transfer takes place, leading to the reduction of nitro groups. Finally, the aniline product is released from the catalyst surface, leaving the catalytic site free to follow another cycle. The reaction between the nitrobenzene molecule and the adsorbed BH_4_^¯^ ions, along with the Pd NP can be suggested as the rate-determining step.

### Catalyst recyclability

**Figure 11:**
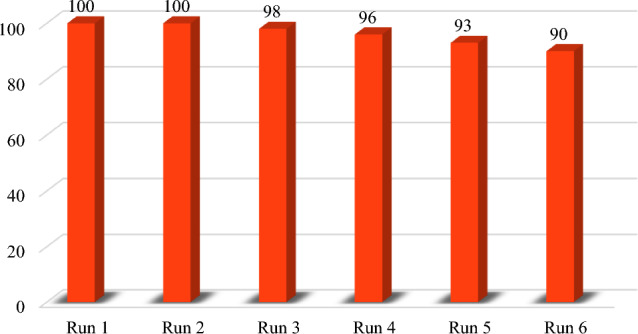
The recyclability of Pd@Her-TCT-PDA nanocatalyst in different reactions.

The used NaBH_4_ plays an significant role in the reaction; because in addition to the hydrogenation of nitro group to amine, it can convert the formed Pd(II) ions to Pd(0) in the catalyst. Therefore, the excess amounts of NaBH_4_ is essential for reusability of the catalyst to have Pd(0) on the catalyst. Due to the recyclability significance of a catalyst for several times, the used Pd@Her-TCT-PDA MNPs was recycled and re-catalyzed the model nitrobenzene reduction for six cycles. First of all, we collected the used catalyst (0.4 g), and washed it with hot EtOH and warm water for removal of the adsorbed organic and inorganic compounds. Subsequently, it was dried in an oven at 80 °C for 2 h, and then reused it for a subsequent run under the optimized reaction condition. Figure 11 depicts the yield of each cycle, and compared to the first run, only a 10% drop in efficiency was observed in the sixth run. This fact confirms the practical catalytic usage of Pd@Her-TCT-PDA MNPs in chemical reductions.

Moreover, the drop in in catalytic efficiency of Pd@Her-TCT-PDA MNPs after 3rd run is probably due to the presence of different degrees of Pd species on catalyst surface, which means the formed Pd(II) didn’t reduce to Pd(0) even in the presence of NaBH_4_. The possibility of Pd-leaching is very low because using an atomic absorption spectroscopy (AAS), its content was examined in the reaction mixture after separation of the catalyst for each run and no detectable level was observed (detection limit of instrument was 3.0 μg L^−1^). The fixed ligands on the silica surface and strong complexation with Pd species are the main reasons for the not-leaching of Pd into the solution.

### Comparison with literature

A literature survey was accomplished for the comparison of the results obtained from the nitro-reduction through different conditions (Table 3). The loaded Pd nanoparticles on the graphene oxide could reduce the nitro group completely in the presence of NaBH_4_. Salicylic acid acted as the reducing agent in this reaction^61^. Although this is a comparable reaction, its reaction time is higher than this study. The use of carbon nanotube and sodium sulfide is another way for the mentioned reduction, however, its drawback is the activation of nanotube at high temperature and running the reaction under basic conditions^62^. Photocatalytic reduction in the presence of Bi_2_MoO_6_ photocatalyst needs UV-irradiation^63^. The use of zero valent iron for NO_2_-reduction has been recently considered, while it needs longer time to complete the conversion^64,65^. As a conclusion, the loaded Pd on the hercynite magnetic nanoparticles can act as a good reductive catalyst.

**Table 3:** Comparison of studies on nitrobenzene reduction.

Entry	Catalyst	Solvent	Conditions	Yield (%)	Refs.
1	Pd NPs on graphene oxide	EtOH	salicylic acid, NaBH_4_, 3 h, rt	100	^61^
2	Carbon nanotubes	–	Sodium sulfide, pH 8, 400 °C		^62^
3	Bi_2_MoO_6_	MeOH	UV, rt., 1 h	95	^63^
4	H_2_O_2_/HCl-treated Fe(0)	H_2_O	rt., 2 h	95	^65^
5	Biochar supported sulfidated nano zero valent iron	–	dark, 30 °C, 1 h	85	^64^
6	Pd@Her-TCT-PDA	H_2_O	NaBH_4_, 50 °C, 35 min	100	This work

## Conclusion

To have a magnetic, reductive catalyst comprising a palladium complex, hercynite magnetic nanoparticles was initially synthesized through a co-precipitation process of the salts of ferrous and aluminum. Then, its particles were coated with silica, and the silica surface was modified with heterocycles of PDA and TCT. Finally, Pd(II) was loaded on it and reduced to Pd(0) using NaBH_4_. Characterization techniques confirmed that the modification process was successfully performed. The applicability of Pd@Her-TCT-PDA MNPs was tested in the reduction of nitro group of organic compounds to their corresponding amines. This nanocatalyst could give the products in warm water, in brilliant yields within short reduction times.

## Data Availability

All data and materials were provided in the manuscript and readers can contact to corresponding author to receive additional explanation.
